# Paddington Green Children's Hospital

**Published:** 1902-05-10

**Authors:** 


### The Institutional Workshop.

### PADDINGTON GREEN CHILDREN'S HOSPITAL.

The attendance at the annual meeting on May 1st was both representative and numerous, and the chairman, Mr. George Hanbury, contrasted it with justifiable pride with those of other and larger hospitals, when scarcely a dozen people were present. The board-room, where the meeting took place, was tastefully decorated with flowers and plants.

The past year has been an eventful one for the hospital, for it has seen the purchase of a convalescent home at "Fair View," Upton Road, Slough, at a cost of £1,200 The lease, at £75 a year ground rent, has 62 years to run, and includes "Upton House," which is let for the like amount. The sanitary and other improvements that were found necessary involved a cost of upwards of £600. The new home is situated about a mile from Slough Station and has behind it a large garden surrounded by a red-brick wall. Miss Anderson, who for eighteen years has been matron of the hospital, having been compelled to relinquish this post on account of ill-health, has taken up the lighter work of matron of the new convalescent home. Miss S. Biddulph Pinchard succeeds Miss Anderson as matron of the hospital.

Mr. George Hanbury, having taken the chair, Mr. C. W. Empson, the hon. secretary, read the report, from which it appeared that the ordinary income for the year showed a small improvement, there being an increase of nearly £66 in annual subscriptions. The expenditure had, however, more than proportionately risen, the numbers of both in- and out-patients being larger than in the previous year.

The Chairman then moved the adoption of the report, and went on at once to speak of the new convalescent home, and to point out how fortunate they had been in securing a house that was so entirely suitable to them at so moderate a cost. Only a few days ago, said Mr. Hanbury, he had paid a visit to the home, and was delighted to see the children enjoying themselves in the large garden. The home had never been formally opened, but they would be very glad to see any visitors who liked to go down and inspect it.

During the past year the hospital had been closed for a few weeks on account of typhoid fever and measles, and although it was open again now, yet they could not allow strangers to visit on account of the small-pox.

The Chairman pleaded earnestly for help towards the maintenance of the new convalescent home, so that it might not become a drag upon the hospital. The parents of the children paid something towards their support, and both they and their children keenly appreciated the benefits they derived from it; in fact, the only danger was lest the children should become too fond of the home, and be unwilling to leave it.

The finances of the hospital were not in a very satisfactory condition, but then, as the Chairman went on to remark, that was the usual state of things with regard to the finances of all hospitals. The war and the increase in the income-tax had affected everyone and in consequence they were considerably in debt to their bankers. Much had been done by many kind friends in the way of raising money by entertainments, but what the hospital really needed were two or three large legacies. Several had already been promised and solicitors had offered to mention the hospital to their clients, but none had as yet been received. There was no reason why such gifts should not be made during the lifetime of the donor, and indeed this would be a far more satisfactory arrangement.

Sir T. Fowell Buxton, G.C.M.G., who seconded the motion, spoke of the very pleasant impression left upon his mind by a visit to the wards of the hospital. He noticed above all how restful and happy the children appeared to be, and what perfect sympathy there existed between them and their nurses. In fact the whole atmosphere of the place was sympathetic and harmonious; the walls with their pretty and artistic pictures, and the flowers in such rich abundance. There were one or two little ones who could not put a name to the bluebells or daffodils, but that was because they had not been in the hospital long.

The Rev. M. R. Neligan, M.A., Vicar of St. Stephens, Westbourne Park, who was the next speaker, expressed it as his opinion, after reading the report, that the hospital did not receive sufficient support from the districts of Paddington and Kilburn. Out of the 16 collections that had been made on behalf of the hospital, only nine were from local places of worship. It certainly seemed that a greater effort should be made to interest people, and especially children, in the work of the hospital. It was particularly gratifying to notice that the payments made by in- and out-patients amounted to nearly £325, which showed how active an interest they were taking in the work.

Referring to the special cots that had been founded in memory of departed friends and relatives, Mr. Neligan said he considered one of the duties most commonly overlooked by people in this country was that of giving thanks for the dear ones who had been spared to them. What could be more fitting than that the parents of a child who had just been given back to them, after a severe illness, should dedicate a cot in this hospital as a thank-offering?

Votes of thanks to the honorary officers, and to the Chairman, having been passed, the proceedings terminated.

